# A Study of Factors Influencing the Volume of Responses to Posts in Physician Online Community

**DOI:** 10.3390/healthcare11091275

**Published:** 2023-04-29

**Authors:** Jingfang Liu, Yu Zeng

**Affiliations:** School of Management, Shanghai University, Shanghai 201800, China

**Keywords:** online health community, text analysis, linguistic pattern, physician

## Abstract

Today’s diverse health needs place greater demands on physicians. However, individual doctors have limited capabilities and may encounter many unsolvable medical problems. The physician online community provides a platform for physicians to communicate with each other and help each other. Physicians can post for help about problems they encounter at work. The number of responses to physicians’ posts is critical to whether or not the problem is resolved. This study collected information on 13,226 posts from a well-known physician online community in China to analyze the factors that influence the number of post replies. In the analysis of the post content of the physician online community, this study innovatively introduces word usage features in the medical field. TextMind was used to extract the rate of several types of words in posts that frequently appear when describing medical information. Ultimately, we found that the rate of time words, visual words, auditory words, and physiological process words used in posts had a positive and significant effect on the number of post responses. A series of new post features has been found to have an impact on the number of post replies in physician online communities. This finding is beneficial for physicians to quickly obtain peer assistance through online platforms, increasing the likelihood of solving workplace challenges and improving physician care, as well as the success of physician online communities.

## 1. Introduction

Since the beginning of the 21st century, online health communities have been growing with the continuous development of information technology and the increasing concern about health issues. Patients can easily find suitable doctors in online health communities to consult about their conditions for follow-up diagnoses and treatment advice. Online health communities can effectively improve patients’ health self-management [1] and self-care [2]. Moreover, social support from online health communities can significantly improve patients’ quality of life [3,4]. The emergence of online health communities has benefited patients a great deal. Patients use online health communities and trust the information provided by healthcare professionals [5]. However, they often have overly high expectations that doctors can take care of all their illnesses for them. This is coupled with the fact that today the perception of health has shifted from the mere treatment of disease to the ongoing management of health and prevention of disease. In addition, health-related needs are more diverse. These increasing needs and expectations are placing greater demands on many healthcare professionals in terms of consultation services and knowledge reserve.

However, it may be difficult for physicians to respond to these demands and meet all patients’ expectations because of the limitations of the current level of medical care in society and of their own knowledge [6,7]. Doctors’ words have authority in the medical field, and patients can turn to them for help with their illnesses. Yet, who do the physicians turn to when they encounter difficult medical problems? According to previous studies, the priority for physicians who seek help is to turn to other physicians in the same department of their hospital [8], and then to search for online information with the help of the Internet [9]. Meanwhile, the emergence of the physician online community provides another way for physicians to seek help and communicate.

There are even some unique advantages of the physician online community compared to offline help-seeking and Internet searching. First, the greater number and range of doctors accessible in a physician online community increases the likelihood of problem-solving. The users of physician online communities are medical staff with certain medical expertise. They come from different hospitals and departments across the country and may be doctors, medical students, or other practitioners in the medical industry. The emergence of physician online communities allows physicians to exchange expertise across hospitals without geographical barriers or cultural restrictions [10]. When physicians encounter a medical problem, they have an extremely limited offline recourse, but the collective wisdom of the large number of fellow physicians in the physician online community may make the problem more easily solved. 

Second, physician online communities can help solve problems of a particular nature more quickly. Information searched on the Internet is largely general in nature, but it often does not meet the needs of physicians, as the problems they encounter in their work are often unique. The physician online community allows users to post for help, using text or pictures to clearly describe their dilemmas. As long as there is an Internet connection, users can browse and post information in the community whenever they wish and anywhere, which helps to improve the efficiency of user communication. 

Third, the physician online community retains many users’ postings and replies, bringing together the experience and wisdom of peers for later learning and discussion. The information exchanged in the online community is basically public, which provides medical professionals, especially medical students who are new to the profession, with a wealth of cutting-edge information and high-quality knowledge for learning and career development.

However, although physician online community provides a platform for many doctor users to communicate, it also suffers from many of the problems common to online communities. As most users are not familiar with each other, there is limited activity in terms of mutual communication and discussion [11]. So, there are some posts on the platform that do not receive any response. Posters tend to hope that their posts receive more replies, as this way they can get more solutions and can compare and analyze the many responses from other physician users in order to select the most appropriate answer to their problems. However, it is undeniable that there will always be some posts with few replies. Posts that receive no replies or only a relatively small number of replies make it difficult for posters to get better solutions. In short, the number of replies to a post is crucial to the resolution of a doctor user’s problem. On the other hand, an increase in the number of replies to posts also means closer communication between users and an increase in user activity, which all contribute to the prosperity of the physician online community. Therefore, how to elicit more replies to posts is a matter of concern and thought for both physician users and the platform.

## 2. Literature Review

According to the composition of user groups and the purpose of communication, online health communities can be divided into three categories: online patient communities, online doctor-patient communities, and physician online communities [12]. However, the current state of research is that most of the existing literature focuses on the first two types of communities, while research on physician online communities is still very inadequate. In addition, current research in the field of online health communities focuses more on the utility impact of platform services on the recipient side, i.e., patients, but few studies focus on the provider side of health services, i.e., healthcare workers in general [12]. Although there is less emphasis on healthcare professionals in existing studies, it is undeniable that their engagement behaviors are critical to the sustainability of online health communities [13,14].

Although medical professionals are more medically literate than the general population, they still need to acquire more medical information. A study by Magrabi et al. found that physicians conducted an average of 8.7 searches per month to meet their information needs [15]. The clinical information-seeking behavior from the right sources allows physicians to increase their knowledge [16,17], improve communication with colleagues to enhance professional standing and credibility [18], and improve their professional services [19]. Physician online communities provide good platform conditions for information exchange between physician users across geographical and departmental boundaries [12]. Their existence has been shown to facilitate the exchange of information among physicians. Barnett et al. indicated that by using commercial virtual communities, general practitioners can improve knowledge-sharing and overcome geographical boundaries, essentially addressing professional and structural segregation [20]. In addition, Bientzle et al. conducted an experiment with medical students by building an open-source platform that provided opportunities to chat and upload documents and found that students were highly motivated to use the online platform and that the online peer counseling and collaborative atmosphere on the platform were very positive. In addition, students showed a significant increase in content knowledge and more confidence in their knowledge compared to before online peer counseling [21].

There is a wide variety of user discussions in the physician online community. By analyzing the content of relevant posts in the “Forensic Occupational Therapy” discussion group, Dieleman and Duncan identified six purposes for which members use online discussion groups: seeking and providing advice, networking, requesting and sharing material resources, service development, defining the role of occupational therapists, and student learning [22]. Peng et al. used discussion threads from an online medical forum to analytically explore the complaints of medical students, residents, and faculty in Canada and the United States and revealed three key themes: the challenges inherent in the hierarchical and demanding nature of medicine; the need to safeguard well-being; and a culture that tolerates unprofessional behavior [23]. Kathleen et al. found that the topics often discussed by nurses in online nursing forums were issues of career planning and clinical skills [24].

The communication among users in physician online communities is often accompanied by the spontaneous information sharing behavior. Physicians’ online information sharing behaviors have received extensive attention from researchers. Yang et al. developed an empirical model based on self-determination theory and Maslow’s hierarchy of needs theory. They finally concluded that personal motivation (including reputation and monetary rewards) and social motivation (doctor-patient interaction) all positively contribute to physicians’ knowledge contributions in the online health community, and that physicians’ professional status plays a moderating role in this [13]. Zhou et al. studied the factors influencing online volunteering behavior among health professionals based on motivation theory. The result showed that intrinsic motivation (technical competence) and extrinsic motivation (online reputation and financial rewards) can promote their voluntary knowledge sharing behavior, while the interaction term of intrinsic and extrinsic motivation can negatively affect voluntary sharing behavior [12]. 

In studying how to improve the efficiency of information transfer in physicians’ online communities, Li et al. collected the interaction data of the top 102 physicians active in the neurology column of the Lilac forum. These interaction data were used to construct an interaction network. By analyzing the network structure, Li et al. gave some suggestions to improve the information exchange between doctors in the e-health community and to promote the service level of the platform [25]. Rooderkerk and Pauwels looked at the content of posts on Innovations in Health, a LinkedIn discussion group for healthcare professionals, and found that posts with the following characteristics had a higher number of comments. They were readable, controversial, practical, written by authors with high social or expert status, did not contain hyperlinks, and were not written on weekends [26].

By reviewing the literature, we found that there are few studies in the field of physician online communities and few studies analyzing the factors influencing the number of post responses in communities from the perspective of post content. The existing literature summarizes the influencing factors mainly regarding the subjective perception and experience of reading the posts in terms of content, but less regarding the aspect of wording in the post texts. Previously, Jiang et al. showed that the use of perceptual words and health-related vocabulary in patients’ texts in online health community can have an impact on the social support they receive [27]. Similar studies have shown that how words are used in textual representations is also important [28,29]. Therefore, this study takes into full consideration the characteristics of physician online communities and innovatively introduces several words commonly used in the medical field into post analysis. This enables exploration and discovery of the impact of a series of wording features on posts and the number of responses received by posts in physician online communities.

## 3. Research Hypothesis and Research Model

In physician online communities, users convey information through text and images. The length of text is closely related to the number of words in the post, which is the most striking feature of the post. The length of the post often reflects the amount of information carried in the post, but too long a message can have a detrimental effect. According to previous studies, lengthy text may prevent other users from understanding the information sought by the posters [30]. And the higher reading burden may also reduce the enthusiasm of other users to communicate with them [30]. Considering that users of physician online communities are medical workers who log in and participate in the platform after a busy and stressful workday, short and concise posts may be more acceptable to them when they want to participate in the discussion. Therefore, this paper proposes the following hypothesis.

**Hypothesis** **1:** *In the physician online community, the shorter the text length of the post, the more replies it receives.*

A study by Peter et al. indicated that the use of pictures closely related to written or oral narratives can significantly increase the attention and memory of health education information and improve comprehension compared with the use of words alone [31]. The pictures in physician online communities, such as examination reports and photos of adverse reactions, often convey visual information that is difficult to represent in text. Moreover, these pictures can help other users better understand the current situation or problem faced by the poster. Posts with pictures may be more appealing to other users compared to text-only posts, thus making them more interested in participating in the discussion of the post and establishing a connection with the poster. Therefore, it is reasonable to assume that:

**Hypothesis** **2:** *In the physician online community, if the post contains pictures, it will receive more replies.*

It has been demonstrated that the use of punctuation is related to the popularity of posts and the number of comments [32]. Question marks can show extreme tension in text [33]. COVID-19 video titles with interrogative pragmatic expressions are more popular with viewers [34]. If a request is phrased as an explicit question with a “?”, it is more likely to receive a useful response [35]. The question mark clearly identifies the location of the question in the post, which allows other users of the physician online community to notice the poster’s question at a glance. A higher percentage of posts using question marks may also mean that posters are more eager to seek help. This has a greater emotional impact on potential respondents who come to view the posts, which may further entice these potential respondents to join the discussion rather than leave quickly. On this basis, Hypothesis 3 was formulated:

**Hypothesis** **3:** *In the physician online community, the post with a higher rate of question marks will receive more replies.*

Posts can often express the emotions of the poster. In the past, there were many studies on text emotion. By analyzing COVID-19 tweets, one study found that topics with negative emotional polarity attracted more replies and favorites than positive tweets [36]. In academic social networking sites, questions containing linguistic features such as sadness, positive emotions, and second-person pronouns have a positive effect on response volume [37]. And in online cancer support groups, messages with highly positive emotions were less likely to receive responses [38]. These studies have different subjects and different findings. In the domain of physician online communities, the use of positive emotion words and negative emotion words may both stimulate the browsing user to react and respond emotionally. So, the following two hypotheses are put forward:

**Hypothesis** **4:** *In the physician online community, the post with a higher rate of words expressing positive emotions will receive more replies.*

**Hypothesis** **5:** *In the physician online community, the post with a higher rate of words expressing negative emotions will receive more replies.*

In the medical field, temporal information has proven to be useful in clinical research progress [39,40]. Almost all types of electronic case records contain temporal information as an important indication of clinical information for disease treatment [41,42]. In the medical field, temporal information helps physicians in their diagnostic and treatment decisions [43]. It is essential to clearly record the status of a patient’s condition at each meaningful time [44]. For example, as time passes, a patient’s temperature, blood pressure, etc., may change constantly. More time words in physician online communities may more clearly describe patient information over time, which helps users in the community to better grasp the details of the patient’s condition and derive treatment options, which may facilitate responses from users. Therefore, the following hypothesis is proposed.

**Hypothesis** **6:** *In the physician online community, the post with a higher rate of time words will receive more replies.*

Lin et al. analyzed the linguistic patterns of medical students’ reflective writing and coded words such as emotional, visual, auditory, sensory, and physiological, and found that all of these types of words were adequately used in their writing [45]. Looking, listening, questioning, and feeling the pulse are the four basic methods of diagnosing diseases in Chinese medicine. Thus, the conditions obtained from visual, auditory, and feeling are information that often needs attention in medicine. In physician online communities, many of the posts by users are related to medicine and pharmacology. The content of these topics often cannot avoid the description of the physiological process, such as describing the physiological state of a case, introducing contraindicated conditions or side effects of medication, etc. So, physician online communities may use words about visual, auditory, feeling, and physiological processes more commonly compared to other online communities. These types of words may describe the relevant medical conditions more clearly and in detail, thus contributing to the participation of other medical users in the discussion, and the following hypotheses are proposed.

**Hypothesis** **7:** *In the physician online community, the post with a higher rate of words denoting vision will receive more replies.*

**Hypothesis** **8:** *In the physician online community, the post with a higher rate of words denoting auditory concerns will receive more replies.*

**Hypothesis** **9:** *In the physician online community, the post with a higher rate of words indicating feeling will receive more replies.*

**Hypothesis** **10:** *In the physician online community, the post with a higher rate of words indicating physiological processes will receive more replies.*

**Figure 1:**
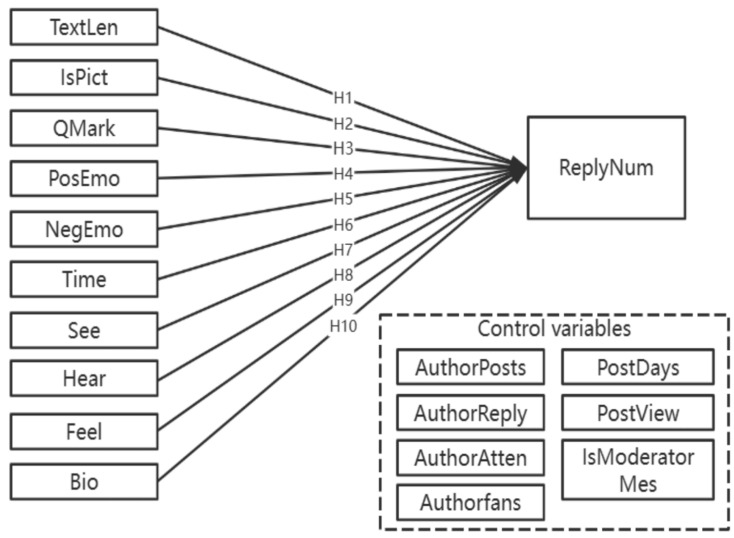
Research model of post response volume in physician online communities.

Based on the above assumptions, the research model of this paper is proposed, as shown in Figure 1.

In the research model in Figure 1, the dependent variable is ReplyNum, which indicates the number of replies to a post. There are 10 independent variables, among which TextLen and IsPict denote the text length of the post and whether the post contains any picture, respectively. QMark indicates the proportion of question marks in the post, which expresses the syntactic characteristics of the post. PosEmo and NegEmo indicate the proportion of positive and negative emotion words in the post, which expresses the emotional wording characteristics of the post. Time, See, Hear, Feel, and Bio denote the rate of time words, visual words, auditory words, feeling words, and physiological process words in the posts, respectively. All five variables describe the wording characteristics of the posts, and all five categories of words involved are closely related to medical care.

In addition, this study also considered other factors that may affect the number of post replies: AuthorPosts (total number of posts by posters), AuthorReply (total number of replies by posters), AuthorAtten (number of other users followed by posters) and Authorfans (number of followers of posters), PostDays (number of days between the posting date and the collection date), PostView (the number of views of the post), and IsModeratorMes (whether the post was commented on by a moderator). These seven factors were also important in influencing the dependent variable, but they were not the focus of this study (this study focuses on the textual content characteristics of the posts), so the above seven variables were included in the control variables.

This paper will test the 10 previously mentioned hypotheses and discuss the effect of control variables on the dependent variable.

## 4. Materials and Methods

### 4.1. Data Source

The data selected for this study were obtained from a well-known physician online community in China. There are more than 30 professional channels in the online medical forum, covering the main medical professional fields, containing 18 discussion forums and more than 130 boards. The platform was established to facilitate learning and communication among medical professional users in medicine, pharmacy, and research. Anyone is free to register as a user and browse the online medical forum freely. However, in order to ensure a good communication environment for medical professionals in the platform and to prevent patients from entering the platform to post and seek medical treatment, the platform has an audit barrier before users post or reply to posts on the platform. Users need to provide medical licenses, medical student study certificates, or other relevant documents to apply for certification. Moreover, users can only post or reply to posts after receiving approval for certification, which to a certain extent prevents non-medical professionals from joining. Therefore, users of the online medical forum are medical professionals such as licensed physicians, pharmacists, or medical students. This also enhances the interpretability of the data in this study to some extent.

This study collected all public discussion data from November 2020 to November 2022 in the cardiovascular section of the online medical forum. There are two main reasons for choosing the cardiovascular section: on the one hand, from the perspective of forum data, the total content volume (total posts and replies reached about 885,000), the number of followers (about 1,774,000), and the daily updated content volume (the daily updated content volume can generally reach more than 500) of the “Cardiovascular” section are the largest volume in the online medical forum. Medical professional users have a high level of attention and participation in this section. On the other hand, in the real world, cardiovascular disease is the number one cause of death worldwide, and in China, the prevalence of cardiovascular disease is increasing year by year, so it is very important to manage cardiovascular disease [46]. The data we collected includes: posting-related information, such as post title, text content, posters’ ID, posting time, posting views, replies, etc.; poster-related information, such as the total number of posts and replies of the user, as well as the number of followers and fans, etc.; and information generated from interactions between users, such as moderator messages. After eliminating posts with permissions, posts that have been deleted or posts missing important data due to poster cancellation, we eventually obtained 13,226 complete posting data for the follow-up study.

### 4.2. Variable Definition and Measurement

The variables for this study were extracted from the above data. The dependent variable is the number of replies to the post, which is directly represented by the data obtained. Among the control variables, the values of AuthorPosts, AuthorReply, AuthorAtten, Authorfans, and PostView are also obtained in this way. The value of PostDays is obtained by calculating the number of days between the posting date and the data collection date. And IsModeratorMes is a classification variable whose value is determined by dividing the posts into two categories based on whether they have messages left by the moderator. The value of IsModeratorMes is 1 for the category of posts with moderator messages, and 0 for the other category of posts.

Among the independent variables, the value of TextLen is expressed by counting the total number of words in the title and body of the post. This is due to the fact that when observing the data, it was found that 826 posts had indicated the intention of posting in the title, while in the body part only images were added without additional text content. Considering that the title of the post is informative, the sum of the post title and body text length were used as the measure of this variable. IsPict is similar to IsModeratorMes in the way it measures. It also categorizes the posts first and then codes them. The other independent variables were determined with the help of TextMind to generate the results.

TextMind is a more mature lexicon-based Chinese language analysis tool. It has been used in several studies to capture psychological features in text content [28,47]. With the help of TextMind, it is easy to get the frequency of a certain type of word in a text [48]. TextMind provides 102 categories of words to be analyzed [48]. Positive emotion words, negative emotion words, time words, visual words, auditory words, feeling words, and physiological process words that need to be analyzed in this study are among them. If one enters Chinese text in TextMind, it will analyze and output the rate of 102 categories of words in this text through the built-in thesaurus. The eight independent variables, QMark, PosEmo, NegEmo, Time, See, Hear, Feel, and Bio in this study are quantified in this way.

The specific variables and related descriptions are shown in Table 1.

In this study, we refer to Lyu et al.’s study of personal narrative texts [28]. The values of the independent variables: PosEmo, NegEmo, Time, See, Hear, Feel, and Bio were calculated based on the rate of relevant words in the posts. By using TextMind, we quantified the use of these categories of words above in the text data of each post from the point of view of word usage.

## 5. Results

### 5.1. Descriptive Statistical Analysis

The information related to each variable was extracted from the 13,226 posting contents, and the results of the obtained descriptive statistical analysis are shown in Table 2.

IsPict and IsModeratorMes are 0–1 categorical variables, while all other variables are continuous variables. ReplyNum has a maximum value of 724 and a minimum value of 0. This means that the highest number of replies received by posts reached 724, but there were still some posts without any replies. Although every post has a number of views, there is a big gap between the highest and lowest views. The total posts, total replies, number of followers and number of fans of posters also differed greatly among individuals. In addition, the mean values of IsPict and IsModeratorMes are 0.338 and 0.059, respectively, which indicates that the number of posts without images is higher than the number of posts with images, and that most of the posts do not receive messages from moderators.

### 5.2. Correlation Analysis

To avoid the problem of multicollinearity, this study examined the two-by-two correlation coefficients between the core explanatory variables and other explanatory variables (control variables) for a total of 17 variables. The specific results of the correlation analysis of the 17 variables are shown in Table 3.

From Table 3, we find that the maximum value of correlation coefficient between the variables is 0.554, and the two corresponding variables are AuthorReply (total number of replies by posters) and AuthorAtten (number of other users followed by posters), and both are control variables. In addition, the values of other correlation coefficients between the variables were less than 0.5. None of the two correlation coefficients between the 17 variables exceeded the threshold value of 0.7. This indicates that there is not a strong correlation between the independent and control variables.

In addition, calculating the variance inflation factor (VIF) for the independent and control variables is also a common method used in multicollinearity testing. The specific estimates obtained are shown in Table 4. The maximum VIF was 1.67, and the minimum VIF was 1.01, while the VIF for all variables were well below the recommended threshold level of 10. Meanwhile, the mean value of VIF is 1.14, which is close to 1 and corroborates the results of the correlation coefficient in Table 3, further indicating that multicollinearity is not a critical issue in this study [49].

### 5.3. Hypothesis Testing

By analyzing the data of this study, the variance of the dependent variable ReplyNum is much larger than the mean, and the values are non-negative integers. Furthermore, considering that there are many zero values in this data, the zero-inflated negative binomial regression model is chosen for estimation. The specific analysis results are shown in Table 5.

Hypothesis 1 states that the shorter the length of the post text, the higher the number of replies received by the post. The results in Table 5 show that the number of replies is negatively correlated with the length of the text content, which is statistically significant (β = $-1.41\times 10^{-4}$, *p* < 0.001). For each additional word in the post text, the number of replies decreased by 0.01%. This suggests that posts with concise content are more likely to be responded to by other users in physician online communities. Hypothesis 1 is supported.

IsPict is a categorical variable and according to the results in the table, the dependent variable ReplyNum has a significant positive correlation with it (β = 0.705, *p* < 0.001). The regression coefficient of this variable is positive; therefore, posts that contain images receive a higher number of responses than posts that do not contain images, and hypothesis 2 is supported.

The results in the table show that the regression coefficients of QMark are positive and show significance at the 0.001 level. So, there is a significant positive relationship between the rate of question marks appearing in the text of a post and the number of replies received to that post (β = 2.107, *p* < 0.001). The use of question marks in posts is common in physician online communities where many users come with the purpose of solving medical problems. The results of the hypothesis testing in Table 5 indicate that posts using a higher rate of question marks are more likely to receive more responses. Hypothesis 3 is supported.

Hypotheses 4 and 5 state that posts containing more positive emotion words and negative emotion words will receive more responses, respectively. The results show that there is a significant positive relationship between the rate of negative emotion words in posts and the number of responses received by posts (β = 3.032, *p* < 0.001); however, there is no significant correlation between the rate of positive emotion words and the number of responses (β = 0.326, *p* > 0.05). Hypothesis 5 was confirmed, while hypothesis 4 was not supported.

There was also a significant positive relationship between the rate of time words in the text of a post and the number of replies received to that post (β = 1.782, *p* < 0.001). Hypothesis 6 was supported. The use of time words is also common in the description of medical information, such as “chest pain for 3 days”, “1 week after surgery”, and so on. The result suggests that as the time information in the text increases, the number of replies received by the post increases.

The results of the hypothesis testing in Table 5 confirm that the rate of using visual words (β = 2.030, *p* < 0.001), auditory words (β = 6.689, *p* < 0.001), and physiological process words (β = 0.633, *p* < 0.001) in the posts is significantly and positively correlated with the number of replies received to the posts. Hypotheses 7, 8, and 10 are supported. However, the results of the study does not confirm that there is a significant correlation between feeling words and dependent variables (β = −0.185, *p* > 0.05), and hypothesis 9 was not confirmed. The lexicon of visual words in TextMind includes words related to the action of “seeing”, such as “seeing clearly”, as well as words obtained visually, such as “crease” and “rosy”. The auditory words are similar. The lexicon of auditory words includes verbs related to “to hear”, such as “whisper”, and those obtained by hearing, such as “applaud”. The lexicon of feeling words includes words related to the sense of touch, such as “pressure”, words obtained through the sense of touch, such as “hot”, and words related to psychological feelings, such as “comfortable” and “gentle”. Doctors need to see, hear, and touch to assess their patients’ conditions, and physician users need to provide this relevant information in order to communicate about medicine in the physician online community. From the results of the hypothesis testing, it appears that posts using a higher rate of visual words and auditory words in the physician online community receive more responses. Physiological process words such as “fat” and “anti-cancer” also express clear physiological information in the medical field. Posts in physician online communities with higher rates of physiological process words may be more appealing to fellow physicians.

Additionally, some of the control variables were significantly related to the dependent variable. There was a significant positive relationship between PostView and ReplyNum (β = $1.12\times 10^{-4}$, *p* < 0.001), which indicates that the more views a post gets, the more replies the post receives. There was a significant negative relationship between PostDays and ReplyNum (β = $-2.73\times 10^{-4}$, *p* < 0.001). IsModeratorMes had a positive and statistically significant correlation with ReplyNum (β = 0.692, *p* < 0.001), which indicates that posts that were commented on by moderators received more replies than posts that were not commented on. Among several control variables related to posters, there was a significant negative correlation between AuthorPosts and ReplyNum (β = $-1.92\times 10^{-4}$, *p* < 0.001), while there was a significant positive correlation between AuthorReply and ReplyNum (β = $1.46\times 10^{-4}$, *p* < 0.001). This means that the more total posts a poster makes, the fewer replies the post receives. The more total replies a poster makes, the more replies the post receives. In addition, AuthorAtten (β = $-5.04\times 10^{-5}$, *p* > 0.05) and Authorfans (β = $5.32\times 10^{-8}$, *p* > 0.05) were not significantly correlated with the dependent variable. This suggests that users may not care about the information on the posters’ followers and fans when replying to posts. Neither AuthorAtten nor Authorfans have a significant impact on the number of replies to a post.

### 5.4. Robustness Test

To ensure the robustness of the results, two tests were used in this study. Based on the data characteristics that the variance of the dependent variable is much larger than the mean, the results were validated by switching to a negative binomial regression model in the robustness test. In addition, the zero-inflated negative binomial regression model is still built in the robustness test, but only a portion of the original data (the last year of data) is selected. The specific results of the two robustness tests are presented in Appendix A.

By comparing the relevant data, the results of the above two robustness tests remain basically consistent with the regression results of the original model. This indicates that the results are adequately robust.

## 6. Discussion

### 6.1. Main Research Conclusions

This study discusses how the text length, images, and wording of posts in physician online communities affect the number of responses received to posts.

By analyzing the text characteristics of posts, this study found that shortening the text length of posts, using pictures, and using more question marks, time words, negative emotion words, visual words, auditory words, and physiological process words in the post can positively influence the number of responses it received.

Concisely stated, illustrated posts in physician online communities may be more likely to attract responses from other medical professional users. Longer text does not mean higher quality content [50], and longer text is less likely to be understood than shorter text [51]. Reading questions with long texts can also interfere negatively with the quality of responses [30]. Thus, shorter text may be somewhat more advantageous than longer text in terms of getting responses. In addition, it has been shown that pictures can enhance readers’ trust [52], attention, and recall of information [31]. Medical pictures also contribute to patients’ understanding, trust, and recall of medical information [53]. Trust has a significant role in enhancing information interaction [54], so posts with images may have an advantage in obtaining responses.

In previous studies, it was demonstrated that text with question marks was more likely to attract readers’ attention [34]. In this study, we found that the rate of question marks in a post affects the number of replies to a post. We also found that posts containing a higher rate of negative emotion words received more responses, while the rate of positive emotion words in the post had no significant effect on the number of responses. This result differs somewhat from the previous findings, which were verified only in terms of negative emotion words in the post but had different results in terms of positive emotion words [37,38]. This may be because the brain processes negative emotion words more deeply and responds more strongly and persistently than neutral and positive emotion words [55].

This study also found that the use of a higher rate of time words, visual words, auditory words, and physiological process words in posts had a significant positive impact on the number of responses to the posts. All of the above four categories of words are commonly used when expressing medical information. More disclosure of time as well as perceptual aspects may help medical professionals in the physician online community to make medical decisions [43,45]. In a sense, making medical decisions may also contribute to the response to the post behavior of users in the physician online community.

The reason why the relationship between the rate of feeling words in posts and the number of responses to posts has not been confirmed may be that the lexicon of feeling words contains some words related to mental feelings. Such words denote more subjective meanings and run counter to the pursuit of stating objective information in the medical field. And when the post’s feeling words were counted, words related to psychological feelings were also counted. Probably for this reason, hypothesis 9 was not confirmed.

Also, this study found significant relationships between some control variables and dependent variables. First, posts with more views and shorter display time on the platform received more replies. The positive relationship between the number of post views and the number of post replies seems to be easier to explain, but the relationship between the number of days a post is displayed in the community and the number of post replies yields the opposite result from previous studies [56]. This may be because popular medical topics are also changing over time [57] and physician users are keen to discuss fresh topics. For example, COVID-19 was a hot topic of medical discussion in recent years. Before the emergence of COVID-19, the medical hotspot may have been other diseases. 

Second, this study also found that there was a significant negative correlation between the cumulative number of posts made by posters and the number of replies to posts in the physician online community, while there was a significant positive correlation between the cumulative number of replies made by posters and the number of replies to posts. This result may be due to the fact that in online health communities, users who post frequently may be more likely to be perceived in the role of information and emotional support seekers, and users who reply frequently are more likely to be perceived in the role of information and emotional support providers [58]. Users interact with other users by replying to their messages, and the number of replies can also indicate the number of interactions from other users. Based on reciprocity, support providers with more replies in the physician online community are likely to receive more replies from other users. In contrast, the number of posts by a user does not measure the user interaction aspect. Support seekers who post frequently also have a hard time getting more responses to each posting. 

Finally, posts that are commented on by moderators get more replies. On the one hand, this may be because moderators have a higher forum status. Ordinary users may have a parasocial relationship with the moderator [59]. The moderator’s comment on a post is like a “weathervane”, and users are more likely to follow in the moderator’s footsteps and have a discussion under that post. On the other hand, moderators are selective in their comments on posts, and only high-quality posts are selected by the moderators. These posts may also attract more replies because of their high quality.

### 6.2. Contributions of the Research

Our study makes the following theoretical contributions:

First, this study enriches the literature in the area of physician online communities. Previous literature on online health communities has focused more on communication between physicians and patients in doctor-patient online communities, as well as between patients and patients in patient online communities. But little attention has been paid to the communication among physician users in physician online communities. In this study, we selected a well-known physician online community in China as a data source and investigated how the content characteristics of doctors’ posts in the community affected the response volume of posts. Although doctor-patient online communities, patient online communities, and physician online communities all belong to online health communities, they are still very different in terms of user composition and communication content. The discussions in the physician online communities are generally about cutting-edge information in the medical field or the principles of medical diagnosis. The other two types of online health communities (i.e., doctor-patient online communities and patient online communities) do not involve as many medical professionals and do not discuss as much medical information as the physician online communities. This study provides a reference for the research in the field on a specific type of online health community, the physician online community.

Second, in this study, the extraction of the content features of the posts in the physician online community has introduced the words often involved in the medical field. In the past, the research on the posts in this community only considered the characteristics of the subjective feelings of the language through reading. While this study, based on the professional habits of doctors, considers the commonly used words in the medical field, explores and summarizes the impact of these words in posts on the number of replies to posts, and provides new ideas for medical text research.

In addition, this study has important practical implications for both physician users and physician online communities: In terms of physician users, they often participate in physician online communities hoping to have peers join their discussions. The findings of this study can guide physician users to pay attention to how to express themselves in the content of their posts. For example, they may try to use short sections of text, add pictures related to descriptions in the text, pay attention to using more time words, visual words, auditory words, and physiological process words, as well as more negative emotion words and question marks when appropriate. The findings of this study can help users to get more responses and better communication in the physician online community by adjusting the presentation of posting content.

In terms of the physician online community, this study has certain reference implications for their operation and management, such as prompting moderators to leave more messages on posts, which may result in more posts receiving higher response numbers. In terms of designing user posting pages, input boxes for uploading images can be set up specifically, without interfering with users’ activity, with text in prominent places to remind and guide users on how they might phrase their posted text in such a way that may boost response numbers. The platform environment of an online community has a significant impact on users’ information sharing behavior [60]. The interactive feedback from other users also has a positive effect on users’ participation in the community [61,62]. The findings of this study can help guide community administrators on how to better design and operate the platform to further improve user activity and satisfaction and promote thriving physician online communities.

### 6.3. Limitations of the Research

There are still some limitations in this study, which need to be improved in the follow-up study. In this study, only the posts of cardiovascular medicine in a Chinese physician online community were selected as the data source, and the data content was relatively limited to that area. In the future study, the post data of multiple departments will be collected for research, and the posting practices among departments will be compared in order to enrich the research content. The analysis of post content in this study mainly considers aspects of post wording, and in a follow-up study the topics of posts can also be analyzed, which may bring more value to the communication and discussion among medical professional users in the cardiovascular context. In addition, this study mainly analyzed the relationship between the post content characteristics and the post response numbers, and only cross-sectional data were obtained, without considering the dynamic change of post response numbers over time. In the subsequent study, panel data will be used for analysis, and the time factor will be incorporated to ensure the validity of the study results.

## 7. Conclusions

This study examines the factors influencing the number of responses to posts from the perspective of content characteristics of posts in physician online communities and analyzes and validates them with data from a well-known Chinese physician online community. The results show that posts with shorter text, inclusion of images, and more use of negative emotion words, time words, visual words, auditory words, physiological process words, and question marks in the text garner more responses.

Most of the existing studies on online health communities focus on the utility of platform services for the recipients, i.e., patients, and rarely look at the dilemmas faced by the providers, i.e., health care workers. Studies on communication and interaction also focus on communication between doctors and patients, and few studies discuss online communication among doctors. Medical workers in general are the supply side of social medical services, and their development is closely related to everyone’s health, which deserves sufficient attention. This study innovatively introduces the wording characteristics of the medical field into the textual content of posts in physician online communities and finds a series of posting content characteristics that affect the number of responses to posts. The findings of this study can help users in physician online communities acquire more replies and increase their likelihood of finding solutions. It can also provide management suggestions for physician online communities to promote a thriving community by increasing the activity of communication among users.

## Figures and Tables

**Table 1:** Variables and related descriptions.

Variable Name	Variable Type	Variable Description
Dependent variable		
ReplyNum	Continuous variable	Number of replies received for the post
Independent variable		
TextLen	Continuous variable	Total number of words in the title and body of the post
IsPict	Categorical variables	0-No pictures in the post 1-There are pictures in the post
QMark	Continuous variable	The rate of question marks in the post
PosEmo	Continuous variable	The rate of words expressing positive emotions in the post
NegEmo	Continuous variable	The rate of words expressing negative emotions in the post
Time	Continuous variable	The rate of time words in the post
See	Continuous variable	The rate of words denoting vision in the post
Hear	Continuous variable	The rate of words denoting auditory in the post
Feel	Continuous variable	The rate of words denoting feeling in the post
Bio	Continuous variable	The rate of words denoting physiological process in the post
Control variables		
AuthorPosts	Continuous variable	Total number of postings by the poster
AuthorReply	Continuous variable	Total number of replies from the poster
AuthorAtten	Continuous variable	Number of posters following other users
Authorfans	Continuous variable	Number of the poster followed by other users
PostDays	Continuous variable	The number of days between the posting date and the data collection date
PostView	Continuous variable	Total number of the post was viewed
IsModeratorMes	Categorical variables	0-Moderator has no message for this post 1-Moderator has messages for this post

**Table 2:** Summary statistics.

Variable	N	Mean	Std.Dev.	Min	Max
ReplyNum	13,226	6.860	22.987	0	724
TextLen	13,226	678.642	1320.194	4	28,893
IsPict	13,226	0.338	0.473	0	1
QMark	13,226	0.025	0.055	0	1
PosEmo	13,226	0.014	0.034	0	0.5
NegEmo	13,226	0.008	0.018	0	0.5
Time	13,226	0.038	0.045	0	0.571
See	13,226	0.007	0.021	0	0.333
Hear	13,226	0.001	0.007	0	0.333
Feel	13,226	0.002	0.009	0	0.333
Bio	13,226	0.097	0.085	0	1
AuthorPosts	13,226	786.011	2005.954	0	19,000
AuthorReply	13,226	648.372	1894.565	0	15,000
AuthorAtten	13,226	82.268	208.092	0	1262
Authorfans	13,226	9536.510	162,468.100	0	5,858,000
PostDays	13,226	360.291	214.785	25	751
PostView	13,226	1389.818	4954.526	35	137,000
IsModeratorMes	13,226	0.059	0.236	0	1

**Table 3:** Correlation analysis of independent variables and control variables.

Variables	PostView	PostDays	AuthorPosts	AuthorReply	AuthorAtten	Authorfans	IsModeratorMes	TextLen	IsPict	QMark	PosEmo	NegEmo	Time	See	Hear	Feel	Bio
PostView	1.000																
PostDays	0.052	1.000															
AuthorPosts	0.003	0.210	1.000														
AuthorReply	0.262	0.009	0.034	1.000													
AuthorAtten	0.134	0.076	0.031	0.554	1.000												
Authorfans	0.057	−0.012	0.045	0.071	0.047	1.000											
IsModeratorMes	0.139	−0.132	−0.067	0.290	0.123	0.001	1.000										
TextLen	0.099	0.047	0.347	−0.001	−0.050	0.030	−0.028	1.000									
IsPict	0.147	−0.073	−0.121	0.223	0.076	0.042	0.294	−0.013	1.000								
QMark	−0.025	−0.033	−0.066	0.029	0.068	0.072	0.030	−0.123	0.081	1.000							
PosEmo	−0.020	0.010	−0.047	0.004	0.022	0.013	−0.006	−0.070	0.015	−0.054	1.000						
NegEmo	0.005	0.013	0.013	0.002	−0.006	−0.006	−0.001	0.014	0.014	−0.061	−0.011	1.000					
Time	0.013	−0.025	−0.030	0.011	−0.017	−0.006	0.032	−0.006	0.062	−0.108	0.012	0.039	1.000				
See	0.016	0.001	−0.039	0.069	0.032	−0.004	0.035	−0.041	0.089	−0.007	0.035	−0.030	0.004	1.000			
Hear	0.023	0.011	−0.034	0.059	0.011	0.007	0.046	−0.032	0.022	−0.018	−0.010	−0.009	−0.019	0.090	1.000		
Feel	0.013	0.025	−0.014	−0.003	−0.011	−0.003	0.004	0.010	−0.004	−0.038	−0.013	0.056	0.009	−0.022	0.024	1.000	
Bio	−0.011	−0.002	−0.002	0.001	0.024	−0.027	−0.026	−0.089	−0.037	−0.146	0.024	0.054	0.044	0.102	−0.046	−0.020	1.000

**Table 4:** Variance inflation factor of the independent variable and control variables.

Variable	VIF	1/VIF
AuthorReply	1.67	0.599
AuthorAtten	1.47	0.678
AuthorPosts	1.22	0.822
TextLen	1.19	0.839
IsModeratorMes	1.19	0.841
IsPict	1.17	0.858
PostView	1.11	0.903
QMark	1.08	0.924
PostDays	1.08	0.926
Bio	1.06	0.948
See	1.03	0.967
Time	1.02	0.979
Hear	1.02	0.982
Authorfans	1.02	0.983
PosEmo	1.01	0.987
NegEmo	1.01	0.989
Feel	1.01	0.992
Mean VIF	1.14	

**Table 5:** Results of hypothesis testing.

Variable	Coef.	Std. Err.	*p* Value	95% Confidence Intervals	
PostView	$1.12\times 10^{-4}$	$3.50\times 10^{-6}$	0.000 ***	$1.06\times 10^{-4}$	$1.19\times 10^{-4}$
PostDays	$-2.73\times 10^{-4}$	$4.66\times 10^{-5}$	0.000 ***	$-3.64\times 10^{-4}$	$-1.82\times 10^{-4}$
AuthorPosts	$-1.92\times 10^{-4}$	$7.35\times 10^{-6}$	0.000 ***	$-2.06\times 10^{-4}$	$-1.78\times 10^{-4}$
AuthorReply	$1.46\times 10^{-4}$	$6.53\times 10^{-6}$	0.000 ***	$1.33\times 10^{-4}$	$1.59\times 10^{-4}$
AuthorAtten	$-5.04\times 10^{-5}$	$5.72\times 10^{-5}$	0.378	$-1.63\times 10^{-4}$	$6.17\times 10^{-5}$
Authorfans	$5.32\times 10^{-8}$	$5.96\times 10^{-8}$	0.372	$-6.37\times 10^{-8}$	$1.70\times 10^{-7}$
IsModeratorMes	0.692	0.039	0.000 ***	0.616	0.768
TextLen	$-1.41\times 10^{-4}$	$9.33\times 10^{-6}$	0.000 ***	$-1.59\times 10^{-4}$	$-1.23\times 10^{-4}$
IsPict	0.705	0.021	0.000 ***	0.664	0.745
QMark	2.107	0.171	0.000 ***	1.773	2.442
PosEmo	0.326	0.289	0.260	−0.241	0.892
NegEmo	3.032	0.555	0.000 ***	1.945	4.119
Time	1.782	0.211	0.000 ***	1.368	2.197
See	2.030	0.442	0.000 ***	1.164	2.895
Hear	6.689	1.648	0.000 ***	3.460	9.918
Feel	−0.185	1.148	0.872	−2.435	2.065
Bio	0.633	0.118	0.000 ***	0.402	0.864
_cons	0.833	0.028	0.000 ***	0.778	0.888
Sample size			13,226		
0 value sample size			2499		
prob > chi2			0.000		

Note: *** *p* < 0.001.

## Data Availability

Not applicable.

## Appendix A

Both of the following tables show the results of robustness tests.

**Table A1:** Robustness test results (use negative binomial regression model).

Variable	Coef.	Std. Err.	*p* Value	95% Confidence Intervals	
PostView	$1.12\times 10^{-4}$	$8.78\times 10^{-6}$	0.000 ***	$9.51\times 10^{-5}$	$1.30\times 10^{-4}$
PostDays	$-2.73\times 10^{-4}$	$5.71\times 10^{-5}$	0.000 ***	$-3.85\times 10^{-4}$	$-1.61\times 10^{-4}$
AuthorPosts	$-1.92\times 10^{-4}$	$8.55\times 10^{-6}$	0.000 ***	$-2.09\times 10^{-4}$	$-1.75\times 10^{-4}$
AuthorReply	$1.46\times 10^{-4}$	$7.31\times 10^{-6}$	0.000 ***	$1.31\times 10^{-4}$	$1.60\times 10^{-4}$
AuthorAtten	$-5.04\times 10^{-5}$	$8.53\times 10^{-5}$	0.554	$-2.18\times 10^{-4}$	$1.17\times 10^{-4}$
Authorfans	$5.32\times 10^{-8}$	$3.78\times 10^{-8}$	0.160	$-2.10\times 10^{-8}$	$1.27\times 10^{-7}$
IsModeratorMes	0.692	0.040	0.000 ***	0.614	0.771
TextLen	$-1.41\times 10^{-4}$	$1.37\times 10^{-5}$	0.000 ***	$-1.68\times 10^{-4}$	$-1.14\times 10^{-4}$
IsPict	0.705	0.026	0.000 ***	0.654	0.755
QMark	2.107	0.155	0.000 ***	1.803	2.411
PosEmo	0.326	0.288	0.259	−0.240	0.891
NegEmo	3.032	0.627	0.000 ***	1.803	4.261
Time	1.782	0.221	0.000 ***	1.350	2.215
See	2.030	0.566	0.000 ***	0.920	3.140
Hear	6.689	2.129	0.002 **	2.517	10.862
Feel	−0.185	1.221	0.880	−2.579	2.209
Bio	0.633	0.124	0.000 ***	0.390	0.877
_cons	0.833	0.030	0.000 ***	0.774	0.892
Sample size			13,226		
prob > chi2			0.000		
R^2^			0.145		

Note: ** *p* < 0.01; *** *p* < 0.001.

**Table A2:** Robustness test results (using the data of the past year).

Variable	Coef.	Std. Err.	*p* Value	95% Confidence Intervals	
PostView	$9.61\times 10^{-5}$	$4.55\times 10^{-6}$	0.000 ***	$8.72\times 10^{-5}$	$1.05\times 10^{-4}$
PostDays	$-3.79\times 10^{-4}$	$1.08\times 10^{-4}$	0.000 ***	$-5.90\times 10^{-4}$	$-1.67\times 10^{-4}$
AuthorPosts	$-1.43\times 10^{-4}$	$1.12\times 10^{-5}$	0.000 ***	$-1.65\times 10^{-4}$	$-1.21\times 10^{-4}$
AuthorReply	$1.47\times 10^{-4}$	$8.14\times 10^{-6}$	0.000 ***	$1.31\times 10^{-4}$	$1.63\times 10^{-4}$
AuthorAtten	$7.24\times 10^{-5}$	$8.34\times 10^{-5}$	0.385	$-9.10\times 10^{-5}$	$2.36\times 10^{-4}$
Authorfans	$3.16\times 10^{-8}$	$5.11\times 10^{-8}$	0.536	$-6.85\times 10^{-8}$	$1.32\times 10^{-7}$
IsModeratorMes	0.797	0.038	0.000 ***	0.721	0.872
TextLen	$-1.42\times 10^{-4}$	$1.11\times 10^{-5}$	0.000 ***	$-1.64\times 10^{-4}$	$-1.21\times 10^{-4}$
IsPict	0.612	0.025	0.000 ***	0.564	0.660
QMark	2.441	0.181	0.000 ***	2.087	2.795
PosEmo	−0.218	0.338	0.519	−0.880	0.444
NegEmo	4.020	0.635	0.000 ***	2.775	5.265
Time	1.798	0.227	0.000 ***	1.354	2.243
See	1.146	0.529	0.030 *	0.109	2.182
Hear	6.002	1.861	0.001 ***	2.353	9.650
Feel	−1.101	1.424	0.440	−3.892	1.690
Bio	0.657	0.139	0.000 ***	0.385	0.929
_cons	0.863	0.033	0.000 ***	0.798	0.927
Sample size			7403		
0 value sample size			605		
prob > chi2			0.000		

Note: * *p* < 0.05; *** *p* < 0.001.

## References

[1] Van der Eijk M., Faber M.J., Aarts J.W., Kremer J.A., Munneke M., Bloem B.R. (2013). Using Online Health Communities to Deliver Patient-Centered Care to People with Chronic Conditions. J. Med. Internet Res..

[2] Willis E., Royne M.B. (2017). Online health Communities and Chronic Disease Self-Management. Health Commun..

[3] Malinen S. (2015). Understanding user participation in online communities: A systematic literature review of empirical studies. Comput. Hum. Behav..

[4] Audrain-Pontevia A.-F., Menvielle L. (2018). Do online health communities enhance patient–physician relationship? An assessment of the impact of social support and patient empowerment. Health Serv. Manag. Res..

[5] Johansson V., Islind A.S., Lindroth T., Angenete E., Gellerstedt M. (2021). Online Communities as a Driver for Patient Empowerment: Systematic Review. J. Med. Internet Res..

[6] Schuers M., Griffon N., Kerdelhue G., Foubert Q., Mercier A., Darmoni S.J. (2016). Behavior and attitudes of residents and general practitioners in searching for health information: From intention to practice. Int. J. Med. Inform..

[7] Del Fiol G., Workman T.E., Gorman P.N. (2014). Clinical Questions Raised by Clinicians at the Point of Care A Systematic Review. JAMA Intern. Med..

[8] Younger P. (2010). Internet-based information-seeking behaviour amongst doctors and nurses: A short review of the literature. Health Inf. Libr. J..

[9] Bryant S.L. (2004). The information needs and information seeking behaviour of family doctors. Health Inf. Libr. J..

[10] Rolls K., Hansen M., Jackson D., Elliott D. (2016). How Health Care Professionals Use Social Media to Create Virtual Communities: An Integrative Review. J. Med. Internet Res..

[11] Sohn D., Choi Y.-S. (2022). Silence in Social Media: A Multilevel Analysis of the Network Structure Effects on Participation Disparity in Facebook. Soc. Sci. Comput. Rev..

[12] Zhou J., Zuo M., Ye C. (2019). Understanding the factors influencing health professionals’ online voluntary behaviors: Evidence from YiXinLi, a Chinese online health community for mental health. Int. J. Med. Inform..

[13] Yang H., Du H.S., He W., Qiao H. (2021). Understanding the motivators affecting doctors’ contributions in online healthcare communities: Professional status as a moderator. Behav. Inf. Technol..

[14] Stewart S.A., Abidi S.S.R. (2021). Applying Social Network Analysis to Understand the Knowledge Sharing Behaviour of Practitioners in a Clinical Online Discussion Forum. J. Med. Internet Res..

[15] Magrabi F., Coiera E.W., Westbrook J.I., Gosling A.S., Vickland V. (2005). General practitioners’ use of online evidence during consultations. Int. J. Med. Inform..

[16] Schilling L.M., Steiner J.F., Lundahl K., Anderson R.J. (2005). Residents’ patient-specific clinical questions: Opportunities for evidence-based learning. Acad. Med..

[17] Mikalef P., Kourouthanassis P.E., Pateli A. (2017). Online information search behaviour of physicians. Health Inf. Libr. J..

[18] Cook D.A., Sorensen K.J., Wilkinson J.M., Berger R.A. (2013). Barriers and Decisions When Answering Clinical Questions at the Point of Care a Grounded Theory Study. JAMA Intern. Med..

[19] Perley C.M. (2006). Physician use of the curbside consultation to address information needs: Report on a collective case study. J. Med. Libr. Assoc..

[20] Barnett S., Jones S.C., Bennett S., Iverson D., Bonney A. (2012). General practice training and virtual communities of practice-a review of the literature. BMC Fam. Pract..

[21] Bientzle M., Lechner C., Cress U., Kimmerle J. (2019). Online peer consulting for health professionals. Clin. Teach..

[22] Dieleman C., Duncan E.A. (2013). Investigating the purpose of an online discussion group for health professionals: A case example from forensic occupational therapy. BMC Health Serv. Res..

[23] Peng J., Clarkin C., Doja A. (2018). Uncovering cynicism in medical training: A qualitative analysis of medical online discussion forums. BMJ Open.

[24] Abrahamson K., Fox R., Anderson J.G. (2013). What nurses are talking about: Content and community within a nursing online forum. Stud. Health Technol. Inform..

[25] Li Z., Xu X. (2020). Analysis of Network Structure and Doctor Behaviors in E-Health Communities from a Social-Capital Perspective. Int. J. Environ. Res. Public Health.

[26] Rooderkerk R.P., Pauwels K.H. (2016). No Comment?! The Drivers of Reactions to Online Posts in Professional Groups. J. Interact. Mark..

[27] Jiang S., Liu X., Chi X. (2022). Effect of writing style on social support in online health communities: A theoretical linguistic analysis framework. Inf. Manag..

[28] Lyu Y., Chow J.C.-C., Hwang J.-J., Li Z., Ren C., Xie J. (2022). Psychological Well-Being of Left-Behind Children in China: Text Mining of the Social Media Website Zhihu. Int. J. Environ. Res. Public Health.

[29] Zhang W., Zhu W., Nie J., Andrasik F., Blom X.N. (2022). The Effect of Emotion Background on Pathological Internet Users’ Comments on Online News: Evidence from Online Text Analysis. Cyberpsychol. J. Psychosoc. Res. Cyberspace.

[30] Alwin D.F., Beattie B.A., Alwin D.F. (2016). The KISS Principle in Survey Design: Question Length and Data Quality. Sociological Methodology.

[31] Houts P.S., Doak C.C., Doak L.G., Loscalzo M.J. (2006). The role of pictures in improving health communication: A review of research on attention, comprehension, recall, and adherence. Patient Educ. Couns..

[32] Noguti V. (2016). Post language and user engagement in online content communities. Eur. J. Mark..

[33] Plantin C. (2019). Tense Arguments: Questions, Exclamations, Emotions. Informal Log..

[34] Liu J., Lu C., Lu S. (2021). Research on the Influencing Factors of Audience Popularity Level of COVID-19 Videos during the COVID-19 Pandemic. Healthcare.

[35] Tang Y., Hew K.F., Yuan X., Qiao C. (2021). How social instant messaging questions affect replies: A randomised controlled experiment. Behav. Inf. Technol..

[36] Yu H., Yang C.-C., Yu P., Liu K. (2022). Emotion diffusion effect: Negative sentiment COVID-19 tweets of public organizations attract more responses from followers. PLoS ONE.

[37] Li L., Li A., Song X., Li X., Huang K., Ye E.M. (2021). Characterizing response quantity on academic social Q&A sites: A multidiscipline comparison of linguistic characteristics of questions. Libr. Hi Tech.

[38] Lewallen A.C., Owen J.E., Bantum E.O., Stanton A.L. (2014). How language affects peer responsiveness in an online cancer support group: Implications for treatment design and facilitation. Psycho-Oncology.

[39] Pan X., Chen B., Weng H., Gong Y., Qu Y. (2020). Temporal Expression Classification and Normalization from Chinese Narrative Clinical Texts: Pattern Learning Approach. JMIR Public Health Surveill..

[40] Tang B., Wu Y., Jiang M., Chen Y., Denny J.C., Xu H. (2013). A hybrid system for temporal information extraction from clinical text. J. Am. Med. Inform. Assoc..

[41] Kreimeyer K., Foster M., Pandey A., Arya N., Halford G., Jones S.F., Forshee R., Walderhaug M., Botsis T. (2017). Natural language processing systems for capturing and standardizing unstructured clinical information: A systematic review. J. Biomed. Inform..

[42] Rink B., Harabagiu S., Roberts K. (2011). Automatic extraction of relations between medical concepts in clinical texts. J. Am. Med. Inform. Assoc..

[43] Olex A.L., McInnes B.T. (2021). Review of Temporal Reasoning in the Clinical Domain for Timeline Extraction: Where we are and where we need to be. J. Biomed. Inform..

[44] Augusto J.C. (2005). Temporal reasoning for decision support in medicine. Artif. Intell. Med..

[45] Lin C.-W., Lin M.-J., Wen C.-C., Chu S.-Y. (2016). A word-count approach to analyze linguistic patterns in the reflective writings of medical students. Med. Educ. Online.

[46] Xu T., Loban K., Wei X., Wang W. (2022). Determinants of choice of usual source of care among older people with cardiovascular diseases in China: Evidence from the Study on Global Ageing and Adult Health. BMC Public Health.

[47] Liu X., Sun M., Li J. (2018). Research on gender differences in online health communities. Int. J. Med. Inform..

[48] Yuan C., Hong Y., Wu J. (2021). Personality expression and recognition in Chinese language usage. User Model. User-Adapt. Interact..

[49] Mason C.H., Perreault W.D. (1991). Collinearity, Power, and Interpretation of Multiple Regression Analysis. J. Mark. Res..

[50] Fleckenstein J., Meyer J., Jansen T., Keller S., Köller O. (2020). Is a Long Essay Always a Good Essay? The Effect of Text Length on Writing Assessment. Front. Psychol..

[51] Mesmer H.A., Hiebert E.H. (2015). Third Graders’ Reading Proficiency Reading Texts Varying in Complexity and Length: Responses of Students in an Urban, High-Needs School. J. Lit. Res..

[52] FeldmanHall O., Dunsmoor J.E., Tompary A., Hunter L.E., Todorov A., Phelps E.A. (2018). Stimulus generalization as a mechanism for learning to trust. Proc. Natl. Acad. Sci. USA.

[53] Phelps E.E., Wellings R., Griffiths F., Hutchinson C., Kunar M. (2017). Do medical images aid understanding and recall of medical information? An experimental study comparing the experience of viewing no image, a 2D medical image and a 3D medical image alongside a diagnosis. Patient Educ. Couns..

[54] Thiede M. (2005). Information and access to health care: Is there a role for trust?. Soc. Sci. Med..

[55] Wang L., Bastiaansen M. (2014). Oscillatory brain dynamics associated with the automatic processing of emotion in words. Brain Lang..

[56] Wu Y., Ye Q., Li L., Xiao J. (2012). Power-Law Properties of Human View and Reply Behavior in Online Society. Math. Probl. Eng..

[57] Wang T., Huang Z., Gan C. (2016). On mining latent topics from healthcare chat logs. J. Biomed. Inform..

[58] Wang X., Zhao K., Street N. Social Support and User Engagement in Online Health Communities. Proceedings of the International Conference for Smart Health (ICSH).

[59] Tsiotsou R.H. (2015). The role of social and parasocial relationships on social networking sites loyalty. Comput. Hum. Behav..

[60] Xie R., Zhang W. (2022). Analysis of influencing factors of online community knowledge-sharing from the perspective of the platform environment. Kybernetes.

[61] Fang J., Chen L., Wang X., George B. (2018). Not all posts are treated equal: An empirical investigation of post replying behavior in an online travel community. Inf. Manag..

[62] Wang J., Yao T., Wang Y. (2023). Patient Engagement as Contributors in Online Health Communities: The Mediation of Peer Involvement and Moderation of Community Status. Behav. Sci..

